# Putative Prevention of XML Injection Against Myocardial Ischemia Is Mediated by PKC and PLA2 Proteins

**DOI:** 10.3389/fcell.2022.827691

**Published:** 2022-01-24

**Authors:** Ling Jin, Qianqian Yin, Yiqing Mao, Yuanxu Gao, Qing Han, Ruisi Mei, Lixiang Xue, Huanran Tan, Hui Li

**Affiliations:** ^1^ Center of Basic Medical Research, Institute of Medical Innovation and Research, Peking University Third Hospital, Beijing, China; ^2^ Department of Pharmacology, Peking University, Health Science Center, Beijing, China; ^3^ State Key Laboratory of Lunar and Planetary Sciences, Macau University of Science and Technology, Macau, China

**Keywords:** xinmailong injection, myocardial ischemia, network pharmacology, Protein kinase C, PLA 2

## Abstract

**Background:** Xinmailong (XML) injection is a CFDA-approved traditional Chinese medicine with clinical value for heart failure treatment. The present investigation was aimed to evaluate the potential protective roles of this injection on myocardial ischemia and the underlying molecular mechanism.

**Methods:** In our study, we selected two models of myocardial ischemia rats. Rats were randomly divided into six groups, with saline or XML administrated 4 days before ischemia model establishment. ECG of different time intervals and biochemical parameters of end point were measured. The potential mechanisms of the protective role of XML were explored using system pharmacology and molecular biology approaches.

**Results:** Myocardial ischemia rats demonstrated abnormal ECG and serum levels of cTnT. Pretreatment with XML significantly attenuated these damages, especially the medium doses. GO and KEGG analysis revealed that the 90 putative target genes were associated with pathways of fatty acid absorption/metabolism, inflammation, RAAS, and vascular smooth muscle. Further network pharmacology method identified five main chemical ingredients and potential targets of XML injection for myocardial ischemia. Mechanically, the beneficial effect of XML injection was mediated by the reactive oxygen species (ROS) inhibition and inflammation attenuation via regulating the expression levels of targets of PKC and PLA2.

**Conclusion:** These findings indicate that XML exerts protective effects against myocardial injury, with attenuated ROS production, apoptosis, and inflammation. Therefore, we speculate that XML may be an alternative supplementary therapeutic agent for myocardial ischemia prevention.

## Introduction

The cockroach has a long history of the treatment of disease in traditional Chinese medicine. It is bitter in taste and cold in nature, with the component activities associated with heart function enhancement, increased urine amount, and improved microcirculation (Luo et al., 2014). Xinmailong (XML) injection is a bioactive composite extracted from the cockroach that has been approved by the China State Food and Drug Administration (CFDA) in 2006 (2016). XML has been shown to have outstanding curative effects against cardiovascular injury in chronic heart failure owing to its activities of dilating the coronary arteries, antagonizing activation of neuroendocrine systems, increasing blood supply to cardiac muscle, improving cardiac function, and antagonizing ventricular restructuring (Ma et al., 2013; Liu et al., 2014). XML is currently adopted as an optional treatment for chronic congestive heart failure patients in the clinic (Ma et al., 2013; Liu et al., 2014). In addition, XML injection was reported to mitigate epirubicin (EPI)-induced cardiotoxicity *in vivo* (Li et al., 2016).

Myocardial ischemia is a pathological state of the heart that leads to the decrease of oxygen supply to the heart and abnormalities in myocardial energy metabolism, both of which result in further damage to the heart (Heusch, 2016). This disease results in a reduction of the blood flow to the myocardium. A prolonged duration of ischemia can induce myocardial infarction (MI), which is a common cause of heart failure (HF) (Gheorghiade and Bonow, 1998; Tanai and Frantz, 2015). Thus, early intervention in myocardial ischemia should restore blood flow and improve the clinical outcomes for patients with cardiovascular diseases (CVDs) such as HF and MI.

In the present study, we aim to explore whether XML injection is an effective pharmacological intervention for the early treatment of myocardial ischemia. Since Chinese herbal medicines contain multiple active compounds, and each compound can target different genes and proteins, we hoped to identify the key herbal ingredients and therapeutic targets involved in its disease prevention properties.

## Materials and Methods

### Chemicals

XML injection was provided by Tengchong Pharmaceutical Company Limited by shares Yunnan (Yunnan, China). This drug is manufactured in accordance with applicable Good Manufacturing Practice (GMP) and CFDA standards. HPLC analysis was performed to identify its chemical characteristics. Isoproterenol was obtained from Harvest Pharmaceutical Co., Ltd. (Shanghai, China). Propranolol was purchased from Sigma-Aldrich Co. (St. Louis, MO, United States).

### Animals

Adult male Sprague–Dawley (SD) rats, weighing 160–180 g, were purchased from the Animal Center of the Peking University Health Science Center (Beijing, China). Animals were housed in light-controlled and air-conditioned rooms for 7 days for adaptation before experiments. Standard laboratory chow and water were provided ad libitum. Animal experiments were performed in accordance with the “Guidelines for Animal Experiment” and approved by the Animal Care Committee of the Peking University Health Science Center.

SD rats were randomly selected into groups that received normal saline or three different concentrations of XML injection or propranolol (10 mg/kg) every day for a total of 4 days. 30 min after the final administration, two types of *in vivo* myocardial ischemia models were induced. The rats were either subcutaneously injected with isoproterenol (5 mg/kg) or their left anterior descending coronary artery was ligated, while control rats were not (Wu et al., 2011). Heart myocardial ischemia was assessed using the noninvasive cardiac imaging technology of echocardiography (ECG) and by measuring the serum levels of cardiac troponin T (cTnT) biomarker.

### Electrocardiographic Recordings

Surface electrocardiographic (ECG) recordings were obtained from rats that were anesthetized with sodium pentobarbital. For the ECG analysis, the onsets and offsets of the P, Q, R, S, and T waves were determined by measuring the earliest (onset) and the latest (offset) times from lead II.

### Network Pharmacology-Based Analysis

Published literature and the BATMAN-TCM databases were used to identify XML ingredients. Active ingredients were identified using SwissADME, while candidate targets for each ingredient were predicted using the SwissTargetPrediction database. Disease-associated target prediction was performed using DisGeNET, Therapeutic Target Database (TTD), and OMIM databases. The relevant protein–protein interaction networks were extracted from the Human Protein Reference and STRING databases. The Kyoto Encyclopedia of Genes and Genomes (KEGG) pathway database and Gene Ontology (GO) map were used to analyze the disease-related targets and pathways. Finally, an ingredient-target-pathway network was constructed using Cytoscape software (version 3.8.2) to provide a systematic overview of potential target genes and mechanisms for XML action.

### Reverse Transcription and Real-Time PCR

Heart tissue was homogenized and lysed with Trizol reagent (Invitrogen, United States) according to the manufacturer’s instructions. Extracted RNA samples were transcribed into cDNA using the RevertAid RT Reverse Transcription Kit (Thermo, United States). Amplifications were performed with the ROCHE LightCycler 480 Real-Time PCR with the primers listed in Table 1, with the conditions of initial denaturation at 95°C for 2 min followed by 40 cycles of 95°C for 15 s and 60°C for 1 min. Gene transcript abundance levels were normalized to those for GAPDH.

**TABLE 1:** Primers used for quantitative real-time RT-PCR.

Gene	Primer
PRKCA	F: 5′-CCC​AGA​AGC​AAG​CAC​AAG​TT-3′
	R:5′-GACATTGATCACGCACTGCT-3′
PRKCE	F: 5′-ACG​GTG​GAG​ACC​TCA​TGT​TC-3′
	R: 5′-TTG​CAG​TGA​CCT​TCT​GCA​TC-3′
MAPK3	F: 5′-GGC​CCG​AAA​CTA​CCT​ACA​GT-3′
	R: 5′-TCC​AGC​TCC​ATG​TCA​AAG​GT-3′
PLA2GA4A	F: 5′-TTA​ACC​TGC​CGT​ATC​CCT​TG-3′
	R: 5′-CTT​CAA​TCC​TTC​CCG​ATC​AA-3′
CYBA	F: 5′-CAT​GTG​GGC​CAA​CGA​ACA​G-3′
	R: 5′-CAC​TGT​GTG​AAA​CGT​CCA​GCA​GTA-3′
CYBB	F: 5′-TGA​TCC​TGC​TGC​CAG​TGT​GTC-3′
	R: 5′-GTG​AGG​TTC​CTG​TCC​AGT​TGT​CTT​C-3′
GAPDH	F: 5′-ACA​AAG​TGG​ACA​TTG​TTG​CC-3′
	R: 5′-AAA​CAT​GGT​GGT​GAA​GAC​GC-3′

### Measurement of Serum Cardiac Troponin T (cTnT) Levels

Blood was collected from the orbital venous of rats 2 hours after isoproterenol administration or coronary artery ligation. Serum was collected by centrifugation, and the cTnT content was quantified using a double-antibody sandwich ELISA.

### Western Blot Analysis

Heart tissue was homogenized and tissue protein was quantified using the Bradford method. Sixty micrograms of protein was loaded onto an SDS-PAGE gel, separated, and transferred onto a PVDF membrane (Millipore, United States). Antibodies for Bcl-2 (CST, United States), Bax (CST, United States), and GPADH (CST, United States) were diluted 1:1000 and incubated with the membrane overnight at 4°C. The next day, the membrane was washed and incubated with secondary antibodies and then visualized using a luminescence ChemiDoc XRS (Bio-Rad, United States).

### Statistical Analysis

Results are shown as mean ± standard error. Differences between the control and experimental groups were evaluated by one-way ANOVA. *p* < 0.05 were considered to be statistically significant.

## Results

### XML Pretreatment Improves Response to Myocardial Ischemia

To explore whether XML injection could be an effective pharmacological intervention for myocardial ischemia, two different types of animal models were established. The first is the isoproterenol-induced myocardial ischemia rat model (Viswanadha et al., 2021), and the second is the acute myocardial infarction rat model induced by the ligation of the left anterior descending coronary artery (Li et al., 2021).

**FIGURE 1:**
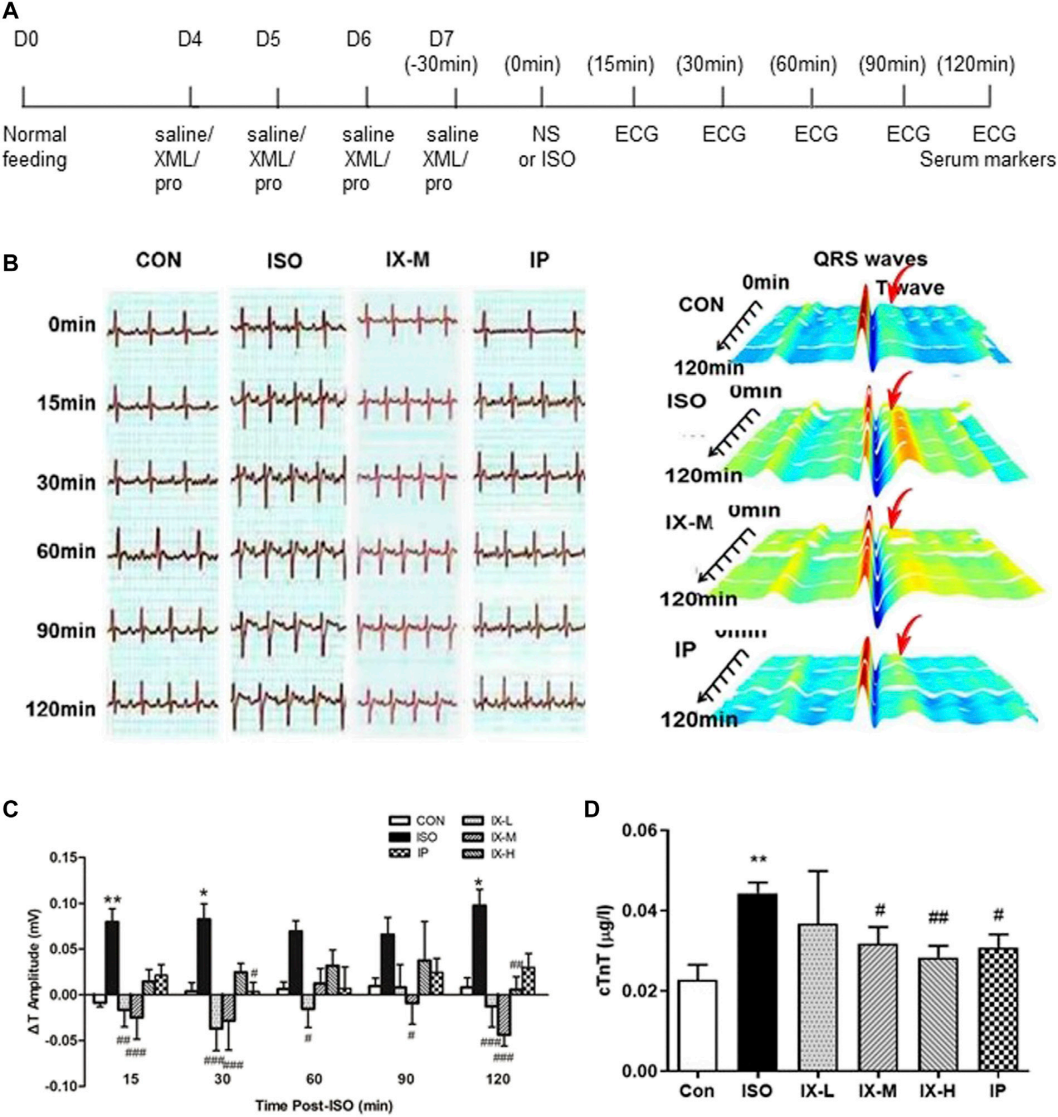
Protective effect of XML on isoproterenol-induced myocardial ischemia in a rat model. **(A)** Timeline of drug administration. CON rats (*n* = 5) were treated with normal saline, while test groups received an XML injection (low dose—L (*n* = 5), medium dose—M (*n* = 6), high dose—H (*n* = 5)), or propranolol (10 mg/kg) (*n* = 5) for a total of 4 days 30 min after the final administration, rats were subcutaneously injected with isoproterenol (5 mg/kg) to induce the myocardial ischemia model. Heart contractile function was assessed using the noninvasive cardiac imaging technology of echocardiography (ECG) and by measuring the levels of the cardiac troponin T (cTnT) biomarker. **(B)** Electrocardiogram recordings of SD rats. **(C)** T interval of rats in each group. **(D)** cTnT levels for each group. Asterisk (*) refers to statistical significance in comparisons with the CON group (*p < 0.05, **p < 0.01), while # refers to comparisons with the ISO group (^#^ *p* < 0.05, ^##^ *p* < 0.01,^###^ *p* < 0.005).

Isoproterenol-induced myocardial ischemia is a classical model used to screen for the cardioprotective effects of pharmacological interventions (Allawadhi et al., 2018). In our study, six groups of SD rats were generated: CON (saline pretreatment for 4 days followed by normal saline injection, *n* = 5), ISO (saline pretreatment for 4 days followed by isoproterenol injection, *n* = 5), IX-L (31.25 mg/kg XML pretreatment for 4 days followed by isoproterenol injection, *n* = 5), IX-M (62.5 mg/kg XML pretreatment for 4 days followed by isoproterenol injection, *n* = 6), IX-H (125 mg/kg XML pretreatment for 4 days followed by isoproterenol injection, *n* = 5), and IP (propranolol pretreatment for 4 days followed by isoproterenol injection, *n* = 5) (Figure 1A). Electrocardiograph (ECG) is the most common test used in the clinic to assess suspected or known cases of myocardial ischemia (Fabiszak et al., 2021). Changes in the ST segment and T-wave should be observed if myocardial ischemia has occurred (Thygesen et al., 2018). Our study showed significant alterations in the ECG patterns in ISO-induced rats compared to normal CON rats (Figure 1B). ECG recordings showed significantly longer T intervals in the ISO rats (Figure 1C). Low and medium doses of XML injection to ISO rats significantly reduced the changes in T intervals, while the medium and high doses of XML reduced the prolonged time for heart rate (Figure 1C, Supplementary Figure S1). Serum cTnT levels have been considered to be a specific marker for myocardial damage, with increases in levels supporting the involvement of myocardial dysfunction (Cullen et al., 2017). cTnT levels were significantly increased in the treatment groups compared with the CON group, with pretreatment with medium and high doses of XML injection reducing the increases in cTnT levels (Figure 1D). These findings suggest that XML pretreatment attenuated cardiac injury induced by isoproterenol exposure, especially for the medium dose.

**FIGURE 2:**
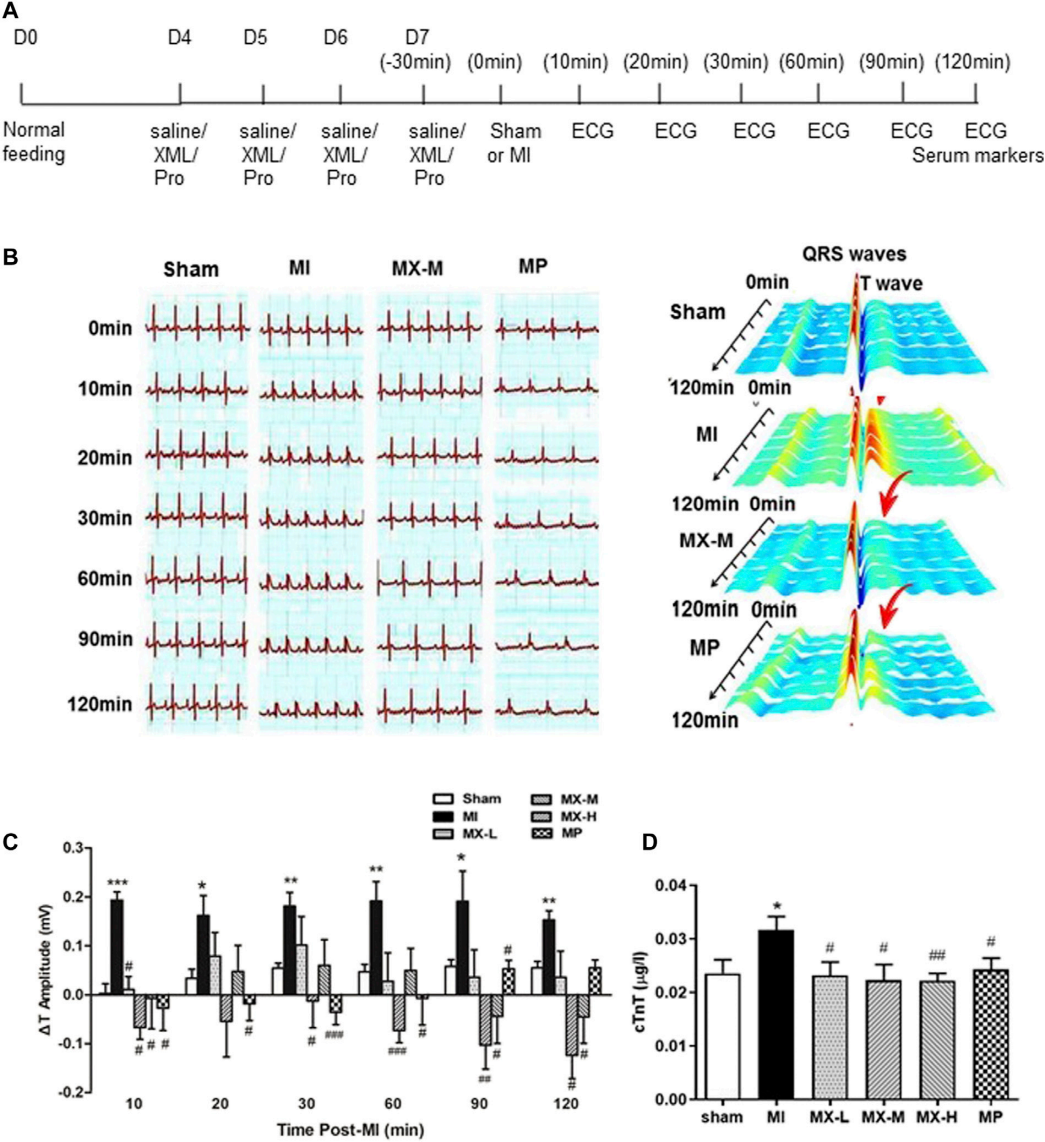
Protective effect of XML on myocardial ischemia induced by ligation of the left anterior descending coronary artery in the rat. **(A)** Timeline of drug administration. Rats received a pretreatment with normal saline (*n* = 4) or XML injection (low dose—L (*n* = 5), medium dose—M (*n* = 6), high dose—H (*n* = 5)), or propranolol (10 mg/kg) (*n* = 6) for a total of 4 days. The left anterior descending coronary artery of rats was ligated 30 min after the final administration to induce the myocardial ischemia model. Heart contractile function was assessed by the noninvasive cardiac imaging technology of echocardiography (ECG) and measurement of serum cardiac troponin T (cTnT) biomarker levels. **(B)** Electrocardiogram recordings of SD rats. **(C)** T interval of the rats in each group. **(D)** cTnT levels for each group. Asterisk (*) refers to statistical significance in comparisons with the CON group (*p < 0.05, **p < 0.01, ***p < 0.005), while # refers to comparisons with the MI group (^#^ *p* < 0.05, ^##^ *p* < 0.01,^###^ *p* < 0.005).

For the second myocardial ischemia model, the left coronary artery was ligated, which should lead to a variable degree of left ventricular myocardial ischemia (Okninska et al., 2021). As above, in this study, our SD rats were also assigned into six groups: Sham (saline pretreatment for 4 days following sham, *n* = 5), MI (saline pretreatment for 4 days followed by coronary artery ligation, *n* = 4), MX-L (15.625 mg/kg XML pretreatment for 4 days followed by coronary artery ligation, *n* = 5), MX-M (31.25 mg/kg XML pretreatment for 4 days followed by coronary artery ligation, *n* = 6), MX-H (62.5 mg/kg XML pretreatment for 4 days followed by coronary artery ligation, *n* = 5), and MP (propranolol pretreatment for 4 days followed by coronary artery ligation, *n* = 6) (Figure 2A). After ligation of the coronary artery, heart function was assessed by ECG and measurement of serum cTnT levels. Ml rats showed significantly longer T intervals and higher cTnT levels (Figures 2B–D), which is consistent with previous findings that coronary occlusion causes an immediate cessation of aerobic metabolism in the ischemic myocardium and durations of coronary occlusion exceeding 60–90 min is considered to be irreversible (Ilic et al., 2021). XML pretreatment significantly altered the upregulation of ΔT amplitude and cTnT level, especially for the MX-M group. For this model, a decreased heart rate was observed in each group with the prolonged time; however, XML injection had no effect on it (suppl. Figure 2). The above data indicate that XML administration for 4 days has protective activity against myocardial ischemia induced by coronary artery ligation.

### Network Pharmacology Analysis of XML

**FIGURE 3:**
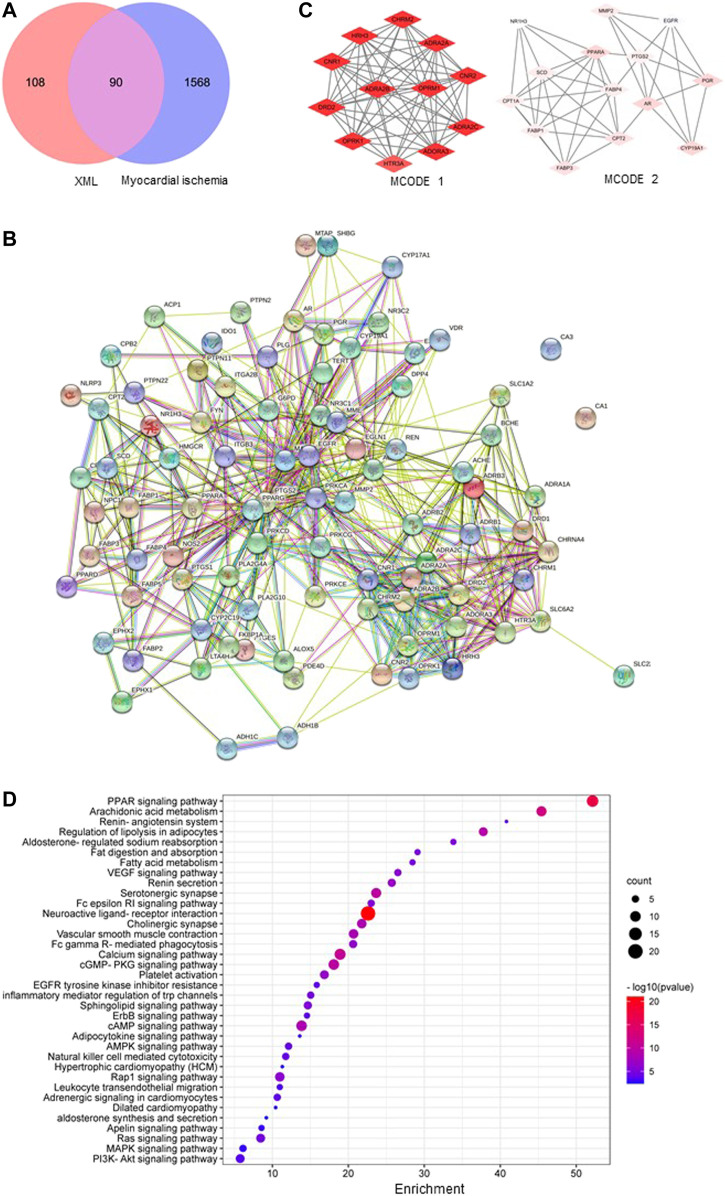
Overlap of genes associated with XML treatment and myocardial ischemia and their functions. **(A)** An intersection generated between genes related to XML-active targets and myocardial ischemia. **(B)** Protein–protein interaction network of the overlapped genes for XML and myocardial ischemia. **(C)** The top 2 MCODEs from the PPI network. **(D)** Most enriched KEGG pathways for XML and myocardial ischemia overlapped genes.

To elucidate the molecular mechanisms of the beneficial effects of XML injection on myocardial ischemia, we first used the network pharmacology method. Published literature and the BATMAN-TCM database were used to identify XML ingredients, and the active components were screened using the SwissADME database. A total of 17 ingredients with high levels of GI absorption and drug-likeness were identified in XML injection (Table 2). From these ingredients, a total of 198 targets, with duplicates removed, were predicted by the Swiss Target Prediction database (Figure 3A). Potential targets for myocardial ischemia were screened using the GeneCards, OMIM, and DisGeNET databases. In total, 1658 gene targets were identified after duplicates were removed (Figure 3A). A Venn diagram was created to visualize the overlap in genes related to active XML ingredients and myocardial ischemia (Figure 3A), which identified 90 intersecting genes.

**TABLE 2:** Basic information on the active compounds in XML injection.

No	Chemical composition	Molecular formula	PK GI absorption	Drug likeness Number
XML1	4-(2-amino-1-hydroxyethyl)-2-benzenediol	C8H11NO3	High	4
XML2	3-hydroxy-4-(N,N,N-trimethyl)-butyric acid	C7H15O3N	High	3
XML3	2,3-butanediol	C4H10O2	High	3
XML4	Pentanoic acid	C5H10O2	High	3
XML5	2-[(2-aminoethyl) amino]-ethanol	C4H12N2O	High	3
XML6	2-piperidine ketone	C5H9NO	High	3
XML7	Dihydro-5-(1-hydroxyethyl)-2(3H)-furanone	C6H10O3	High	3
XML8	catechol	C6H6O2	High	3
XML9	5-oxo-2-pyrrolidinecarboxylic ethyl ester	C7H10NO3	High	3
XML10	1-isopropylcyclobutyl methylamine	C8H16N	High	3
XML11	2-methyl-3-vinyl cyclopentene carboxylic acid	C7H8O2	High	3
XML12	3-pyrrolidin-2-yl-propionic acid	C7H13NO2	High	3
XML13	2,5,5-trimethyl-1,3-cyclohexanedione	C9H14O2	High	3
XML14	Hexadecanoic acid	C16H32O2	High	3
XML15	Hexadecanoic acid, methyl ester	C17H34O2	High	2
XML16	Tridecanoic acid, 12-methyl-, methyl ester	C15H30O2	High	3
XML17	11-Hexadecanoic acid, methyl ester	C17H32O2	High	3

The 90 intersected genes were analyzed using STRING software, with a coefficient of 0.4 indicating correlation (Figure 3B). A PPI network should facilitate our understanding of the regulatory roles of targets. The importance of the functional module in the PPI network is described by the MCODE score (Supplementary Figure S3), and we named the two largest as MCODE1 (containing 64 nodes) and MCODE2 (containing 43 nodes) (Figure 3C). The biological processes of these top 2 MCODEs include “adenylate cyclase–modulating G protein–coupled receptor signaling pathway,” “regulation of catecholamine secretion,” “blood circulation,” and “fatty acid transport,” all of which were strongly associated with the pathological mechanisms of myocardial ischemia (Table 3).

**TABLE 3:** Main biological processes of the top 2 MCODE gene functions.

GO	Description	Log10(p)
GO: 0007188	Adenylate cyclase–modulating G protein-coupled receptor signaling pathway	−14.2
GO:1903531	Negative regulation of secretion by cell	−14.2
GO:0050433	Regulation of catecholamine secretion	−13.3
GO:0008015	Blood circulation	−12.4
GO:0015908	Fatty acid transport	−11.9

To further examine the functions of the 90 overlapping target genes, GO and KEGG enrichment analyses were carried out. As shown in Figure 3D, pathways related to fatty acid absorption/metabolism, inflammation, RAAS, and vascular smooth muscle were identified, including the terms “PPAR signaling,” “arachidonic acid metabolism,” “renin-angiotensin system,” “regulation of lipolysis in adipocytes,” “aldosterone-regulated sodium reabsorption,” and “VEGF signaling pathway”. The GO map contains the top molecular functions of “oxidoreductase activity” and “protein kinase C activity,” and the top biological processes of “circulatory system process,” “lipid localization and transport,” and “inflammation response” (suppl. Figure 4).

**FIGURE 4:**
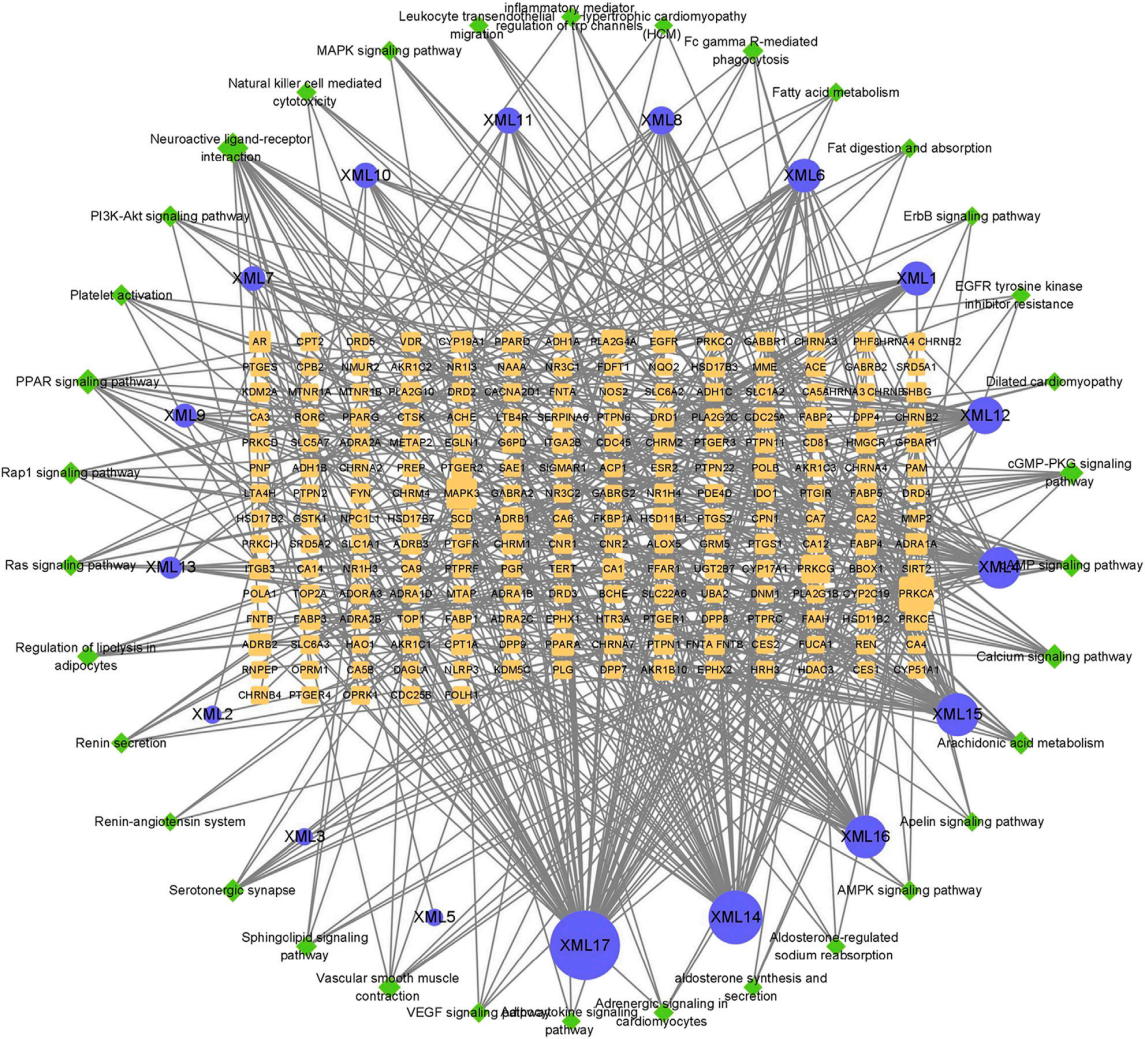
XML ingredient-target-pathway network. Blue circles represent active components, yellow rectangles represent target genes, and green diamonds represent pathways. The size of a node represents its degree. The larger the area, the more important the node.

Network pharmacology can analyze the relationship between drugs, targets, metabolic pathways, and diseases by constructing a network model (Hopkins, 2008). Based on the target and pathway analyses, an entire ingredient-target-pathway network was constructed (Figure 4). In this network, we identified the top five chemical ingredients in XML injection, which were “11-hexadecenoic acid, methyl ester,” “hexadecanoic acid,” “tridecanoic acid, 12-methyl-, methyl ester,” “hexadecanoic acid, methyl ester,” and “pentanoic acid” (Table 4). In addition, we screened for the top ten genes based on their degree values as potential core targets (Table 5) and speculate that these genes might contribute to the molecular mechanisms by which XML injection attenuates myocardial damage.

**TABLE 4:** Putative main compounds in XML injection associated with prevention of myocardial ischemia.

Molid	Chemical name	Degree	Betweenness Centrality	Closeness Centrality
XML17	11-Hexadecanoic acid, methyl ester	74	0.43112	0.44345
XML14	Hexadecanoic acid	51	0.16506	0.35953
XML16	Tridecanoic acid, 12-methyl-, methyl ester	36	0.04302	0.33514
XML15	Hexadecanoic acid, methyl ester	36	0.04302	0.33514
XML4	Pentanoic acid	35	0.06230	0.32203

**TABLE 5:** Putative targets of XML injection in the prevention of myocardial ischemia.

Target	Degree	Betweenness Centrality	Closeness Centrality
PRKCA	25	0.08158	0.35235
MAPK3	19	0.04513	0.34163
PRKCG	17	0.01314	0.29025
PLA2G4A	10	0.01579	0.29581
ADRB1	9	0.01387	0.29440
EGFR	8	0.02173	0.27784
PRKCE	8	0.00354	0.26361
HSD11B1	8	0.06382	0.38775
PPARA	8	0.02203	0.35336
ADRB2	8	0.00997	0.29231

### Putative Involvement of Protein Kinase C (PKC) and PLA2 (Phospholipase A2 Group IVA) in the Prevention of Myocardial Ischemia by XML Injection

Clinically, myocardial ischemia is caused by the blockage of the coronary artery and is defined as an interrupted supply of blood to the left ventricle (Kompa and Summers, 2000). The surgical ligation of the left coronary artery animal model is more similar to human myocardial ischemia (Seong et al., 2021); thus, we evaluated gene changes in this rat model.

Based on the results of network pharmacology analysis (Figure 4; Table 5), first, the top four genes (PRKCA/MAPK3/PRKCG/PLA2G4A) and PRKCE (an isoform of the large PKC family) were picked up for validation in the animal model of surgery-induced myocardial ischemia.

**FIGURE 5:**
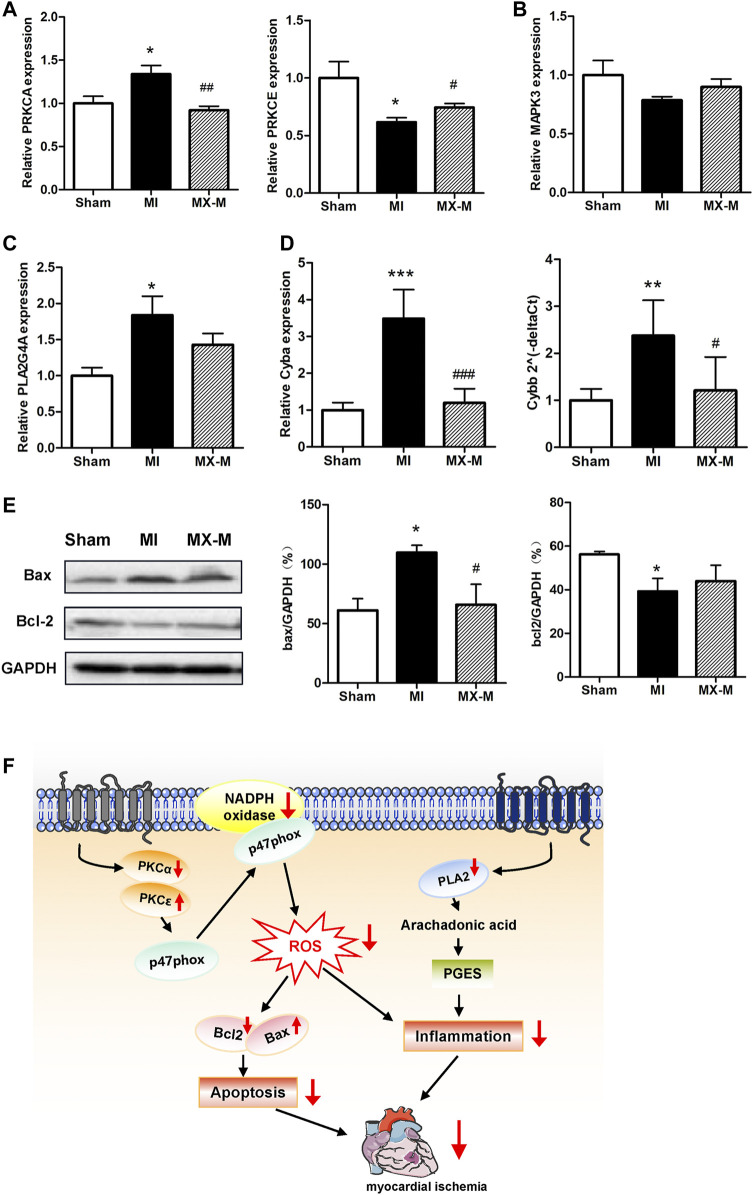
Mechanisms of the protective effects of XML injection on myocardial ischemia. **(A)** Relative PRKCA and PRKCE transcript levels in the Sham (*n* = 5), MI (*n* = 4), and MX-M (*n* = 6) groups. **(B,C)** Relative MAPK3 **(B)** and PLA2G4A **(C)** transcript levels in the Sham (*n* = 5), MI (*n* = 4), and MX-M (*n* = 6) groups. **(D)** CYBA and CYBB gene transcript abundance in the Sham (*n* = 5), MI (*n* = 4), and MX-M (*n* = 6) groups. **(E)** Abundance of Bax and Bcl-2 proteins in the Sham, MI, and MX-M groups (*n* = 3). **(F)** Schematic model for the molecular mechanisms associated with the protective effect of XML injection against myocardial ischemia. Asterisk (*) refers to statistical significance in comparisons with the CON group (*p < 0.05, **p < 0.01, ***p < 0.005), while # refers to comparisons with the EPI group (^#^ *p* < 0.05, ^##^ *p* < 0.01,^###^ *p* < 0.005).

In our study, the expression level of heart PRKCA was enhanced, while PRKCE was reduced upon myocardial ischemia, and their levels returned to normal following the pretreatment of XML injection (Figure 5A). Approximately, 11 isozymes have been identified in the Protein kinase C (PKC) family (Perveen et al., 2021). PKCα is encoded by the PRKCA gene and is the predominant PKC isoform expressed in the heart (Aslam, 2020). Activated or increased PKCα expression is associated with hypertrophy, dilated cardiomyopathy, ischemic injury, and mitogen stimulation (Steinberg, 2012). Another novel PKC isozyme, PKCε, is encoded by the PRKCE gene and is involved in ischemia tolerance following ischemic preconditioning and ischemia injury (Chen et al., 2021). NADPH oxidase is a main downstream protein complex of Protein kinase C activation, and the two mitochondrial function-related indicators of CYBA and CYBB encode the membrane-spanning subunits of this complex (Deng et al., 2012). In the present study, XML pretreatment was shown to mitigate the upregulation of myocardial ischemia induced by CYBA/CYBB levels (Figure 5D).

PLA2 (encoded by the Pla2g4a gene) can hydrolyze phospholipids into arachidonic acid and mediate inflammation (Marsche, 2015). Similarly, XML pretreatment was revealed to significantly lower the ischemia-induced upregulation of PLA2G4A abundance (Figure 5C).

Generally, during ischemia, in addition to the excessive production of ROS and inflammation, programmed cardiomyocyte death (through apoptosis) is also activated (Hotchkiss et al., 2009; Malek and Nematbakhsh, 2015). To clarify how XML injection regulates myocardial cell apoptosis, protein levels for B-cell lymphoma 2 (Bcl2) and Bcl-2–associated X protein (Bax) were measured. Our study revealed that the abundance of Bax and Bcl2 were significantly changed in the MI model and restored in XML pretreated cardiomyopathy (Figure 5E). These results indicate a role for XML in the independent anti-apoptosis mechanism.

Taken together, our study indicates that XML pretreatment augments the abundance of PKCε and suppresses the levels of PKCα, both of which then decrease NADPH oxidase and inhibit apoptosis activity. Meanwhile, reduction of PLA2 levels induced by XML injection results in decreased inflammation (Figure 5F). Thus, the actions of XML injection likely are responsible, at least in part, for the antioxidative, anti-inflammatory, and anti-apoptotic activities via the PKC and PLA pathways. These results closely coincide with our findings from the GO and KEGG analyses and indicate the potential functions of XML injection as an alternative supplementary therapeutic agent for the prevention of myocardial ischemia.

## Discussion and Conclusion

Myocardial ischemia is defined as reduced blood flow to the heart that inhibits heart muscle from receiving enough oxygen (Heusch, 2019). It is a leading cause of morbidity and mortality for CVDs, though certain risk factors such as arteriosclerosis and treatments via surgical intervention can be controlled. According to the 2018 universal definition of myocardial ischemia, it is a disease with clinical events that includes myocardial cell death (seen as ischemic symptoms, ischemic electrocardiographic changes, and coronary artery intervention), and myocardial injury (which results in elevations of cardiac troponin levels) (Thygesen et al., 2018).

Early intervention to the blocked vessel is critical for restoring blood flow to the heart muscle and improving clinical outcomes. Suitable preventative medicines for the treatment of myocardial ischemia are still at an early stage of development. XML injection is an optional treatment for chronic congestive heart failure patients in the clinic. Our results, in the present study, show that XML pretreatment for 4 days can protect rats from myocardial ischemia-induced myocardial injury (Figure 1C, Figure 1E, Figure 2C, Figure 2D, Supplementary Figure S1A), indicating its potential for prevention of injury.

The causes of myocardial ischemia are various and include atherosclerosis, blood clots, and coronary artery spasms (Heusch, 2019). Atherosclerosis may result in myocardial ischemia *via* two different identities, acute coronary syndrome (ACS, type 1 MI) and prolonged myocardial oxygen supply–demand imbalance (type 2 MI) (Smit et al., 2020). ACS occurs because of an unstable plaque rupture or endothelial ulceration and is influenced by factors such as inflammation (Casscells et al., 2003) and hyperlipidemia (Lai et al., 2021). Our study showed that among the top ten KEGG pathways associated with XML injection treatment, five were associated with inflammation and hyperlipidemia, including “PPAR signaling pathway,” “arachidonic acid metabolism,” “regulation of lipolysis in adipocytes,” and “fat digestion and absorption” (Figure 3D). It is also reported that atherosclerotic arteries lead to a dysfunctional endothelium in combination with increased α adrenergic receptors (Naghavi et al., 2003), which is consistent with our findings that the pathways “VEGF signaling pathway” and “vascular smooth muscle contraction” were identified in the KEGG analysis Figure 3D). In addition to ACS and type 2 MI, a further cause for myocardial ischemia is coronary artery spasm. The most important causative factor for this pathophysiology is an increased intracellular calcium concentration in combination with elevated calcium sensitivity (Yasue et al., 2008), which is consistent with our identification of changes in the “calcium signaling pathway” with XML injection treatment (Figure 3D).

The human body is a complex regulatory network; therefore, the therapeutic effects of compounds likely affect more than just their direct targets. Traditional Chinese medicine is a comprehensive medical health-care system and has been applied for centuries to prevent or heal diseases by restoring balance. Herbal medicines contain multiple active compounds, with each compound potentially targeting multiple genes or proteins (Hopkins, 2008; Schenone et al., 2013). XML injection has been reported to contain compounds that include polyhydric alcohols, organic acids, alkaloids, and other micro-constituents; however, the active ingredients have not yet been reported. Our study identified five main components associated with myocardial ischemia prevention (Table 4). XML injection, like other traditional Chinese medicine, contains many components and has multi-target effects. Among the five main components, hexadecanoic acid methyl esters are vasoactive and can dilate blood vessels (Lin et al., 2014). In addition, these components can protect against myocardial ischemia by regulating the metabolism of fatty acids and activating numerous signaling pathways (Cheng et al., 2021). In addition, the ingredient-target-pathway network shown in Figure 4 revealed multiple targets and pathways for XML injection and its attenuation of myocardial ischemia disease and indicated multiple pharmacological activities. This suggests that therapeutic medications that interact with multiple targets might be more effective for complex or chronic diseases such as myocardial ischemia.

Based on the integrated-network-pathway and molecular experiment analysis, we identified PKC and PLA2 as potential targets of XML injection for the improved response to myocardial ischemia (Figure 4, Figure 5A, Figure 5C, Table 4). Protein kinase C (PKC) is a group of multifunctional proteins that phosphorylate target proteins that have biological functions involved in redox signaling, oxidative stress, cell apoptosis, and mitochondrial dysfunction (Bright and Mochly-Rosen, 2005). PLA2 (encoded by the Pla2g4a gene) promotes chronic inflammation by inducing the production of free fatty acids and lysophosphatides (Wang et al., 2021). Both pathways are associated with myocardial ischemia (Marsche, 2015). It is suggested that the overproduction of reactive oxygen species, intracellular calcium overload, and inflammatory cell infiltration are the most important features of myocardial ischemia injury, all of which could be alleviated by XML injection pretreatment. Of course, in addition to the mechanisms of antioxidant, anti-apoptotic, and immune modulation, more accurate mechanisms of XML injection could be identified by high-throughput methods of RNA sequencing, metabolome, and proteome. We will extend this work in near future.

In conclusion, this study revealed the preventative effects of XML injection, a CFDA-approved injection adopted as an optional treatment for chronic congestive heart failure patients in the clinic, for ischemic heart diseases. Pretreatment with XML significantly alleviates the cardiac-linked pathologies, attenuates the release of cTnT, and prevents cardiovascular effects mediated by antioxidant, anti-apoptotic, and immune modulation. Further network pharmacology method analysis identified the five main chemical ingredients, and their potential targets, for XML injection for myocardial ischemia. Our results indicate that XML injection could be used as an alternative supplementary therapeutic agent for the treatment and prevention of myocardial ischemia.

## Data Availability

The original contributions presented in the study are included in the article/Supplementary Material, and further inquiries can be directed to the corresponding authors.
