# Supplementary material for: Putative Prevention of XML Injection Against Myocardial Ischemia Is Mediated by PKC and PLA2 Proteins
Source: Front Cell Dev Biol. 2022 Jan 24;10:827691. doi: 10.3389/fcell.2022.827691 (PMC8819063; doi:10.3389/fcell.2022.827691)
Supplement: Supplementary file 1 [file DataSheet1.PDF]

Supplementary figures

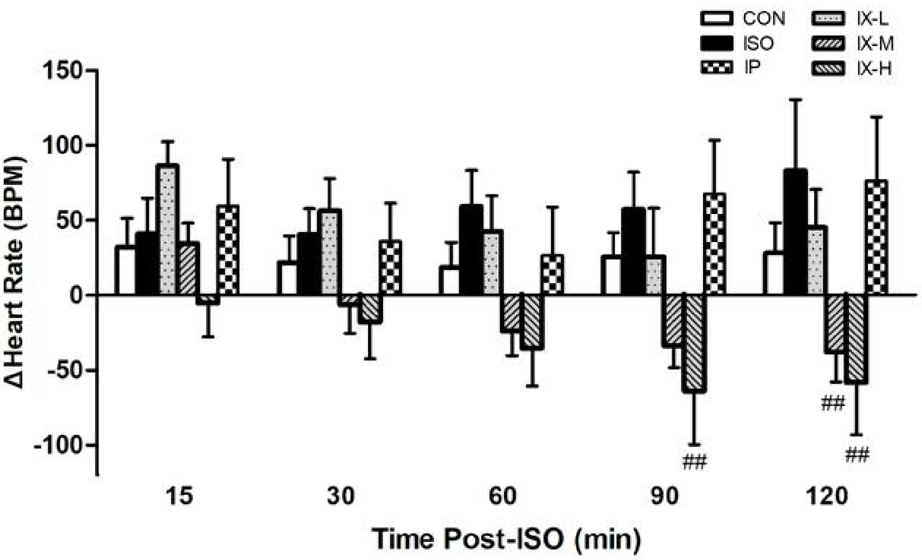

Supplementary figure 1. Changes of heart rate in isoproterenol induced myocardial ischemia rat model. ## refers to comparisons with ISO group ($^{\#}$P < 0.01).

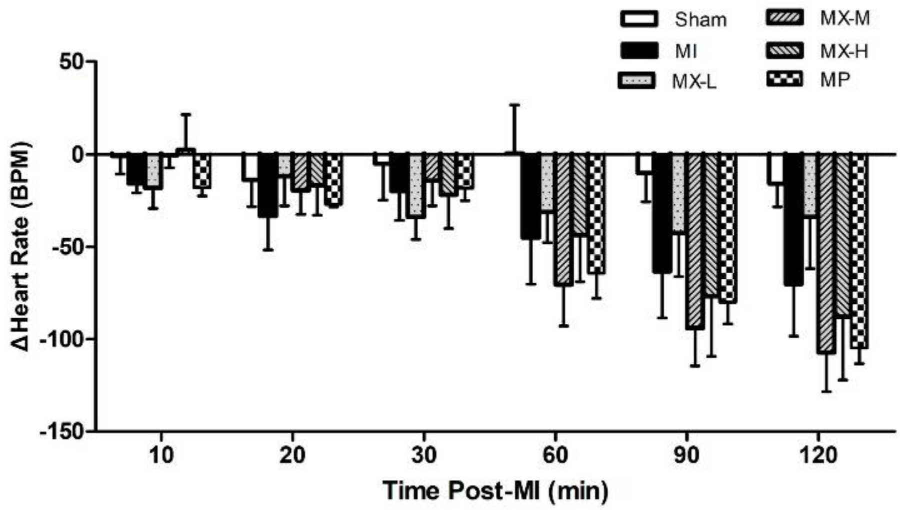

Supplementary figure 2. Changes of heart rate in rat model with the ligation of left anterior descending coronary artery.

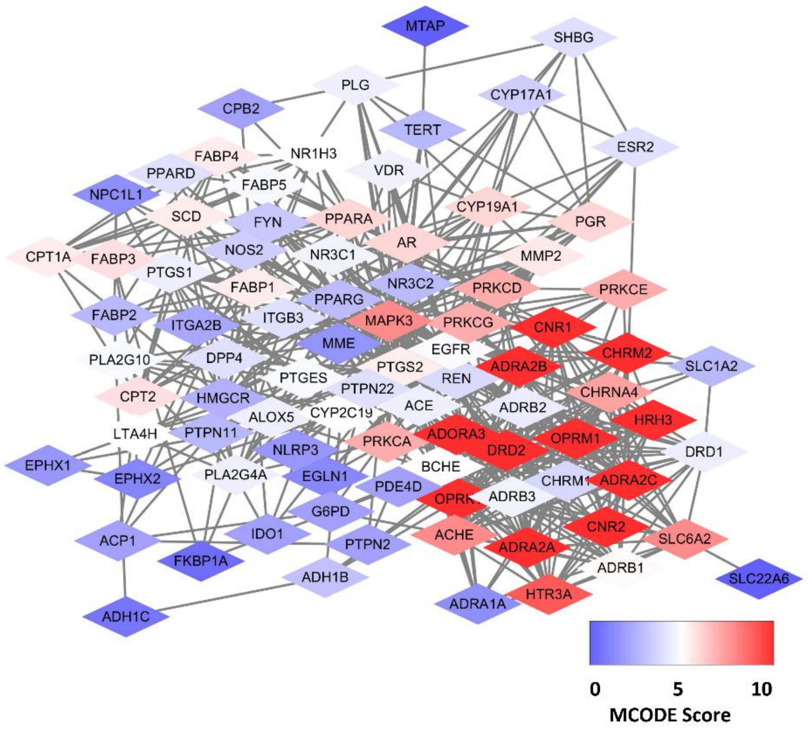

Supplementary figure 3. PPI network display by the color of MCODE score.

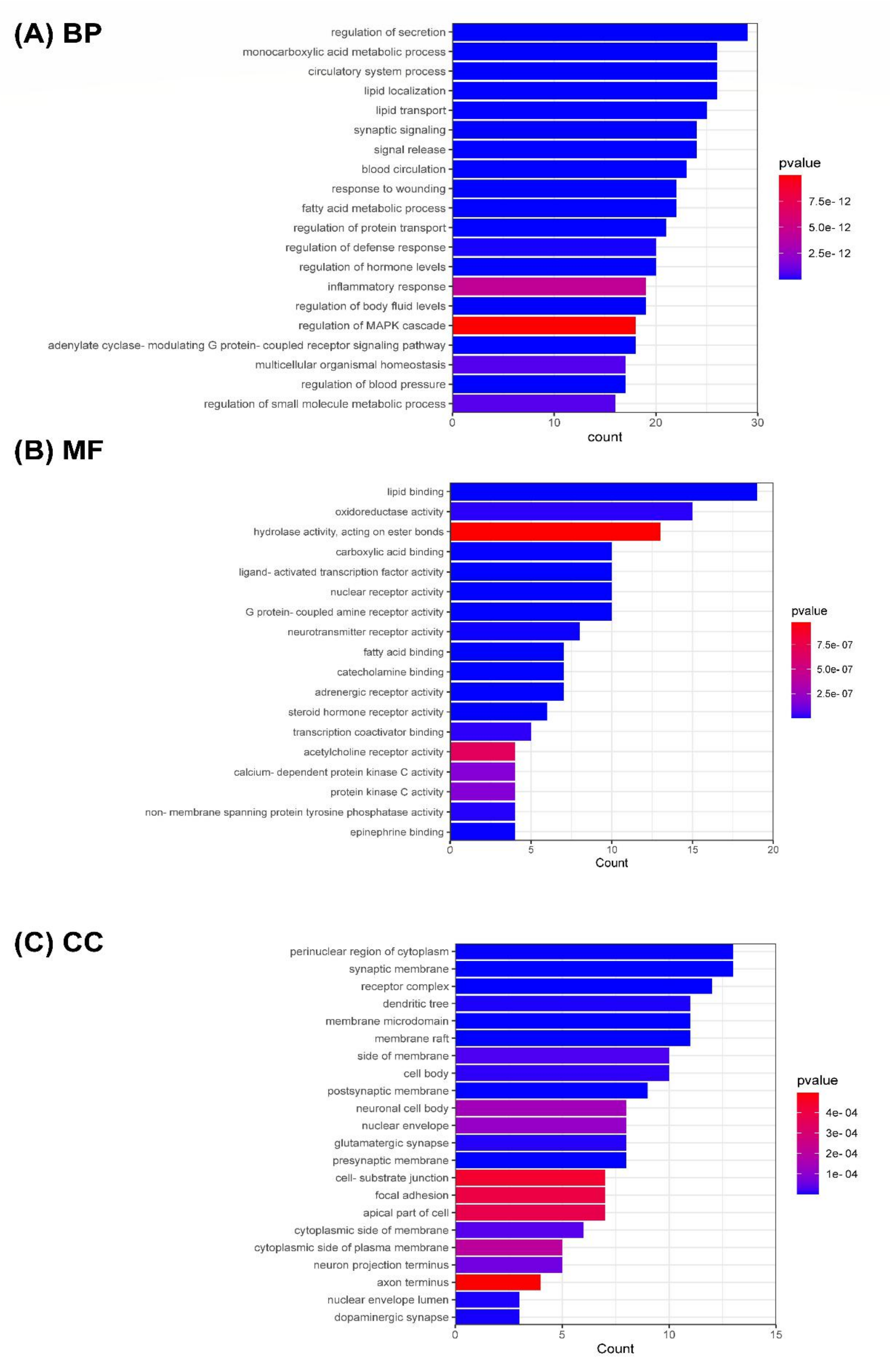

Supplementary figure 4. GO (Gene Ontology) map of putative overlapped target genes. (A) Biological process categories. **(B)** Molecular function categories. **(C)** Cellular component categories.
